# Necrosis from Lactic Acid Injection

**Published:** 1899-04

**Authors:** J. M. Whitney

**Affiliations:** Honolulu, H. I.


### NECROSIS FROM LACTIC ACID INJECTION.

BY DR. J. M. WHITNEY, HONOLULU, H. I.

Being personally acquainted with Dr. William Younger, of San Francisco, and having witnessed his skilful manipulation in the treatment of pyorrhœa alveolaris, I have in many instances adopted his methods and treatment, and in the large number of cases with marked success; but in the following, was it a coincidence or the action of the remedy used that caused the trouble? My attention was first called to question the wisdom of injecting, by means of the hypodermic syringe, lactic acid between the tooth and process by noting the effect upon Mr. K., a teacher aged about fifty years, large and strong, weighing perhaps two hundred and twenty-five pounds. He had been suffering for some time with pyorrhœa alveolaris. I had treated in the usual manner and had restored all the teeth to apparent soundness except the left superior cuspid. There seemed to be left about this, redness of the gums and a little swelling, though no discharge. As he wished to return to his school upon one of the other islands, I injected with the hypodermic syringe a small quantity of lactic acid pure. It caused him great pain, which lasted, with lessening degree, for several hours. In the morning after there was much swelling of the face. Antiphlogistic treatment was at once commenced, but it was not until after several weeks or months had elapsed, when there came away quite a section of process and superior maxillary bone, that full restoration was accomplished. I could not decide whether or not this was caused by the lactic acid or pre-existing conditions.

My next experience was with a Japanese gentleman, who passed through much the same experience as the case just mentioned, though with less pain, this latter being in the lower left first molar. It was not until a section of necrosed process was removed that comfort was restored.

Of these two mentioned I could not be assured but that previous physical conditions might have had something to do with the necrosis found.

The next two were above all suspicion. Rev. Mr. M. was suffering with pyorrhœa alveolaris. All seemed to yield readily to treatment except the lower left cuspid. Around this, as the previous ones mentioned, I injected a small quantity of pure lactic acid. Pain was at once set up, and comfort did not return until a small portion of necrosed process was removed. The last and most serious case was when I injected a small quantity of lactic acid about the anterior root of the first left molar of Miss H. Pain and swelling of the face, as in the other severe cases, were at once attendant. Antiphlogistic treatment being of no avail, I removed the tooth and cut away with the bur all the necrosed process that could be felt, yet the necrosis extended to the second bicuspid, which had to be removed, and, as in the case of the molar, all the roughened process was burred away. To-day, after two weeks from the last operation, I removed a large section of sequestrum, which I hope will bring comfort to the suffering lady. These four unfortunate experiences have taught me to be afraid of lactic acid injected with any force between a diseased tooth and its surrounding process, though I have no fear of applying it by a thin stick of orange-wood or thin platinum carrier. Applied in this manner, in my hands it has proved a most valuable remedy.

